# Downregulation of fibromodulin attenuates inflammatory signaling and atrial fibrosis in spontaneously hypertensive rats with atrial fibrillation via inhibiting TLR4/NLRP3 signaling pathway

**DOI:** 10.1002/iid3.1003

**Published:** 2023-10-27

**Authors:** Yuming Liang, Yun Zhou, Jialin Wang, Yan He

**Affiliations:** ^1^ Department of Cardiology Jiangbin Hospital of Guangxi Zhuang Autonomous Region Nanning China; ^2^ Health Management Center The People's Hospital of Guangxi Zhuang Autonomous Region Nanning China; ^3^ Department of Geriatrics Cardiology First Affiliated Hospital of Guangxi Medical University Nanning China

**Keywords:** atrial fibrillation, atrial fibrosis, fibromodulin, inflammatory signaling, spontaneous hypertension

## Abstract

**Background:**

Myocardial fibrosis is an important factor in the induction and maintenance of atrial fibrillation (AF). Fibromodulin (FMOD) promotes fibrotic gene expression. However, its specific role in spontaneously hypertensive rats (SHR)‐AF remains unclear.

**Methods:**

We analyzed FMOD mRNA and protein expression in rat atrial tissues using RT‐qPCR, Western blot analysis, and immunohistochemistry. Histopathological examination of atrial tissues was performed using hematoxylin and eosin (H&E), Masson's trichrome, and Picrosirius red staining. The levels of inflammatory and fibrosis‐related proteins were measured using Western blot analysis.

**Results:**

FMOD relative mRNA and protein expression levels were notably upregulated in atrial tissues of both AF groups (normal‐AF and SHR‐AF groups) than that in atrial tissues of the no‐AF group (normal and SHR group). This effect was particularly pronounced in the SHR‐AF group. Pathological changes revealed that the extracellular matrix, collagen, collagen fibers, and left atrial diameter were notably increased in the atrial tissues from the SHR‐AF group compared to those in the atrial tissues from the SHR group, whereas the left ventricular fractional shortening and left ventricular ejection fraction were notably lower. Expression of TLR4, MyD88, NLRP3, TGF‐β1, collagen I, and collagen II mRNA were clearly higher in atrial tissues from the SHR‐AF group than in those from the SHR group. Protein levels of TLR4, MyD88, NLRP3, Cleavage‐Caspase‐1, Cleavage‐IL‐1β, TGF‐β1, p‐Smad2, collagen I, and collagen II were clearly higher in atrial tissues from the SHR‐AF group than in those from the SHR group. FMOD knockdown inhibited atrial fibrosis, collagen accumulation, and the TLR4/MyD88/NLRP3 signaling pathway.

**Conclusion:**

Downregulation of FMOD attenuated inflammatory signaling and atrial fibrosis in SHR‐AF by inhibiting the TLR4/NLRP3 signaling pathway. Therefore, FMOD may be a promising therapeutic target in AF.

## Graphical abstract

1. Fibromodulin (FMOD) expression increased in spontaneously hypertensive‐atrial fibrillation rat. 2. Silenced FMOD inhibited TLR4/MyD88/NLRP3 signaling pathway 3. Silenced FMOD alleviates inﬂammatory signaling and atrial fibrosis.

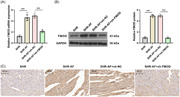

## 1 BACKGROUND

Atrial fibrillation (AF), a persistent arrhythmia commonly encountered in clinical practice, afflicts millions of patients worldwide.^1^ The rising prevalence of AF is a cause for concern, with an estimated 6–12 million Americans projected to be affected by 2050.^2^ In the Chinese adult population, the prevalence of AF has increased from 0.40% to 0.45% between 2012 and 2017 to approximately 1.6% from 2020 to 2021,^3^, ^4^ leading to a significant economic burden.^5^ Its incidence is higher in men than in women.^6^, ^7^ Hypertension, another important public health problem, affects approximately 20–50% of the adult population worldwide, and its prevalence is increasing.^8^ AF is associated with hypertension, and patients with hypertension have a 1.7‐fold higher risk of developing AF than normotensive patients.^9^, ^10^ Myocardial fibrosis plays a crucial role in the induction and maintenance of AF.^11^ Hypertension induces excessive fibroblast proliferation, promotes inflammation, and results in collagen accumulation, further causing diffused fibrosis, left ventricular hypertrophy, and heart muscle remodeling.^12^ However, the causes and basis of hypertension‐induced AF, including the underlying pathogenetic mechanisms, remain unclear.

Fibromodulin (FMOD), coded by a gene located on chromosome 1q32, is an extracellular matrix small leucine‐rich proteoglycan.^13^ FMOD was first reported to be present in the articular cartilage.^14^ Initial research found that FMOD is necessary for tissue repair ^15^ and involved in the regulation of transforming growth factor‐β (TGF‐β) level, inflammation, apoptosis, and metastasis.^16^, ^17^, ^18^ Moreover, FMOD has been associated with certain malignancies such as glioblastoma ^19^ and prostate cancer.^20^ Additionally, FMOD promotes collagen I and α‐smooth muscle actin accumulation to maintain fibrosis in patients with chronic pancreatitis and liver fibrosis.^21^, ^22^ In patients and animals with heart failure, FMOD expression is enhanced in myocardial biopsies, contributing to inflammation and AF.^23^ However, the precise functional roles and mechanisms of FMOD in AF‐associated hypertension in humans have not been investigated.

In this study, we examined FMOD expression in the myocardial tissues of rats with hypertension and AF. We explored the role and mechanism of FMOD in vivo using a spontaneously hypertensive rats (SHR)‐AF rat model.

## 2 MATERIALS AND METHODS

### 2.1 Animals

Specific‐pathogen‐free male normal Wistar Kyoto rats (WKY) aged 14 weeks (normal group, *n* = 10) and SHRs (*n* = 36; weight: 120–150 g; age: 56–62 days) were purchased from Vital River and housed under a 12‐h light/dark cycle at 25°C. All animal experiments were performed in accordance with the National Institutes of Health Guidelines on the Use of Laboratory Animals and approved by the Animal Care and Welfare Committee of Guangxi Medical University (approval No. 201807072).

### 2.2 Establishment of the SHR‐AF model

Caudal artery blood pressure of the rats was monitored, and rats with systolic blood pressure ≥ 160 mmHg and diastolic blood pressure ≥ 90 mmHg were included in the hypertension group. An AF model was established by transesophageal burst rapid pacing, as described in our previous study.^24^ Briefly, the rats were anesthetized by intraperitoneal injection of 3% pentobarbital sodium (40 mg/kg). The stimulation interval was 5 min, and continuous electrical stimulation for 30 s was applied five times at 25, 30, 40, 50, and 83 Hz. Electrocardiograms was recorded simultaneously. If AF rhythms appeared in the SHRs, a rat model of hypertension with secondary AF was considered to be successfully established. Among the 10 normal WKY rats, AF was successfully induced in three normal WKY rats (Normal‐AF group), and seven normal WKY rats remained without AF (Normal group). In the 36 SHRs, AF was successfully induced in 26 rats (SHR‐AF rats) and 10 SHR remained without AF (SHR rat). In the 26 SHR‐AF rats, six rats were used for detecting FMOD expression and 20 rats were used for studying FMOD function. In the 10 SHR rats, five rats were used for detecting FMOD expression and five rats were used for studying FMOD function. One week after AF establishment, the rats were euthanized by intraperitoneal injection of 3% pentobarbital sodium (140 mg/kg) and cardiac tissue samples were harvested for FMOD expression analysis. In addition, sections of cardiac tissue samples were immersed in formalin for histopathological analysis. Finally, three rats (*n* = 3) were randomly selected from each group for western blot analysis.

### 2.3 Quantitative reverse transcription polymerase chain reaction (RT‐qPCR), Western blot analysis, and histopathological examination

RT‐qPCR using *GAPDH* primer sequences and Western blot analysis were performed as described in our previous research.^25^ qPCR assays were performed using a SYBR Green I PCR kit (Takara) on an ABI PRISM 7500 system (Applied Biosystems). FMOD forward (5′‐GCAACAGGATCAATGAGTTCTCC‐3′) and reverse (5′‐GCGCTTGATCTCGTTCCCAT‐3) primers were used. FMOD mRNA was calculated using the $2^{‐\Delta \Delta C_{t}}$ method via *GAPDH* normalization. For Western blot analysis, total proteins were isolated, measured, separated by sodium dodecyl sulfate‐polyacrylamide gel electrophoresis (SDS‐PAGE), and transferred onto PVDF membranes. The membranes were washed and incubated for 4 h at 37°C with primary antibodies, including rabbit anti‐FMOD (K004472P, 1:200 dilution, Solarbio), rabbit anti‐TGF‐β (CST#3711, 1:1000 dilution, CST), rabbit anti‐toll‐like receptor 4 (TLR4, 19811‐1‐AP, 1:1000 dilution, Proteintech), rabbit anti‐myeloid differentiation protein antigen 88 (MyD88, ab131071, 1:1000 dilution, Abcam), rabbit anti‐collagen I (14695‐ 1‐AP, 1:2000 dilution; Proteintech), rabbit anti‐nucleotide‐binding oligomerization domain‐like receptor family pyrin domain containing 3 (NLRP3, NBP2‐12446, 1:250 dilution, NOVUS), rabbit anti‐interleukin 1β (IL‐1β, CST#31202, 1:1000 dilution, CST), rabbit anti‐ Cleavage‐IL‐1β (CST#2021, 1:1000 dilution, CST), rabbit anti‐p‐Smad2 (CST#18338, 1:1000 dilution, CST), rabbit anti‐Smad2 (CST#53391, 1:1000 dilution, CST), rabbit anti‐collagen II (ab307674, 1:1000 dilution, Abcam), rabbit anti‐pro‐Caspase‐1 (ab179515, 1:1000 dilution, Abcam), rabbit anti Cleavage‐caspase‐1‐p20 (TA4005, 1:1000 dilution, Ab‐mark), and rabbit anti‐GAPDH (CST#5174, 1:2000, CST). The membranes were washed and incubated for 4 h at 37°C with horseradish peroxidase‐conjugated secondary goat anti‐rabbit IgG antibodies (1:10000 dilution, ab205718, Abcam). Proteins were detected using an enhanced chemiluminescence kit (Pierce) and quantified using the Image Lab software (Bio‐Rad). For histopathological examination, paraffin‐embedded specimens were sectioned and stained with hematoxylin and eosin (H&E), Masson's, and Picrosirius red, as described in our previous research.^25^

### 2.4 Immunohistochemistry (IHC)

IHC was performed to assess FMOD protein levels in rat atrial tissues. Briefly, formalin‐fixed atrial tissues were embedded in paraffin, and 4 μm sections were cut for IHC analysis. After the serum is blocked, the rabbit anti‐FMOD (K004472P, 1:200 dilution, solarbio) is incubated the sections at 4°C overnight; After wash three times, the horseradish peroxidase‐conjugated secondary antibodies Goat Anti‐Rabbit IgG (1:1000 dilution, ab205718, Abcam) was incubated the sections at room temperature for 50 min; After wash three times, DAB (Servicebio) was incubated the sections at room temperature for 10 min. The positive result is brownish yellow, and the staining is terminated by rinsing the section with tap water. Finally, hematoxylin (Servicebio) was re‐stained the sections for 3 min. The IHC results were analyzed independently by two senior pathologists who were blinded to the outcomes of the study.

### 2.5 Short hairpin (sh) *FMOD* adenovirus construction

Three FMOD‐siRNAs (si‐FMOD‐1: 5′‐GATTACCAGTGACAAGATAGG‐3′, si‐FMOD‐2: 5′‐CTTCTAAGGTCTTAGACAATC‐3′, and si‐FMOD‐3: 5′‐TAAGTCATGACTATCAGTCAC‐3′) were designed. siRNAs were transfected into cultured fibroblasts using Lipofectamine 2000 (Invitrogen), following the manufacturer's instructions. The siRNA interference efficiency was assessed by detecting FMOD mRNA using RT‐qPCR. Sh‐FMOD was synthesized (Genepharma) based on the siRNA sequence with the highest interference efficiency. Negative control shRNAs (sh‐NC, Genepharma) were used as controls. sh‐FMOD and sh‐NC were inserted into the pDC316‐zsGreen1 shuttle plasmid (enzyme cleavage site: *PstI* + *BamHI*) and cotransfected into 293 T cells with the backbone plasmid of Ad. MAX Adenoviral Vector System. Briefly, once 293 T cells reached 80% confluence in 6‐well plates, shuttle plasmid, backbone plasmid, and 8 μL polyfectine agent (Biowit Technologies) were added and allowed to mix. After culturing for 24 h, the cells were transferred to Dulbecco's Modified Eagle's Medium. The transduction efficiency was analyzed using a fluorescence microscope (green fluorescence). Then, 293 T cells were collected, frozen‐thawed three times at −80°C/37°C, and centrifuged at 10,000*g* for 10 min to collect the adenovirus. Finally, the collected adenovirus was transfected into 293 T cells to amplify the virus. After culturing for 48 h, the virus was purified for animal experiments using an adenovirus purification kit (QIAGEN, Dusseldorf). Then the adenovirus‐mediated sh‐FMOD was infected into fibroblasts to analyze whether sh‐FMOD can inhibit FMOD expression in fibroblasts.

### 2.6 FMOD expression interference using adenoviruses in vivo

SHR‐AF (*n* = 18) were randomly divided into three groups: SHR‐AF (*n* = 6), SHR‐AF‐sh‐NC (*n* = 6), and SHR‐AF‐sh‐FMOD (*n* = 6). Based on our prior analysis, 100 μL sh‐FMOD or sh‐NC adenovirus (multiplicity of infection = 1 × 10^10^ pfu/mL) was injected into the jugular vein of SHR‐AF. SHR‐AF rats without injection (SHR‐AF group) were used as controls. Eight weeks after adenovirus injection, the rats were euthanized by intraperitoneal injection of 3% pentobarbital sodium (140 mg/kg) to collect atrial tissues, which were excised for FMOD function and mechanism analysis.

### 2.7 Echocardiography

For echocardiography, rats were anesthetized and placed on an isothermal pad maintained at 40°C. After their chest was shaved, echocardiographic data were analyzed using an Esaote MyLab Seven ultrasound machine (Esaote SpA). Two‐dimensional echocardiograms were obtained from the LV short‐ and long‐axis views. The left atrial diameter (LAD) was measured from the long‐axis view at the aortic valve level to estimate the atrial size using a two‐dimensional echocardiogram. In two‐dimensionally targeted M‐mode images, indices reflecting left ventricular systolic function, including left ventricular fractional shortening (LVFS) and left ventricular ejection fraction (LVEF), were analyzed.

### 2.8 Statistical analysis

All the rats were included in the final analysis. SPSS v.22.0 (IBM Corp.) was used to analyze data, presented as the mean ± standard deviation. Statistical significance was set at *p* < .05. Statistically significant differences between the sh‐NC and sh‐FOMD groups were evaluated using Student's *t*‐test. Differences involving multiple comparisons were assessed using one‐way analysis of variance, followed by post hoc Tukey's tests.

## 3 RESULTS

### 3.1 FMOD expression was upregulated in the SHR‐AF model

**Figure 1:**
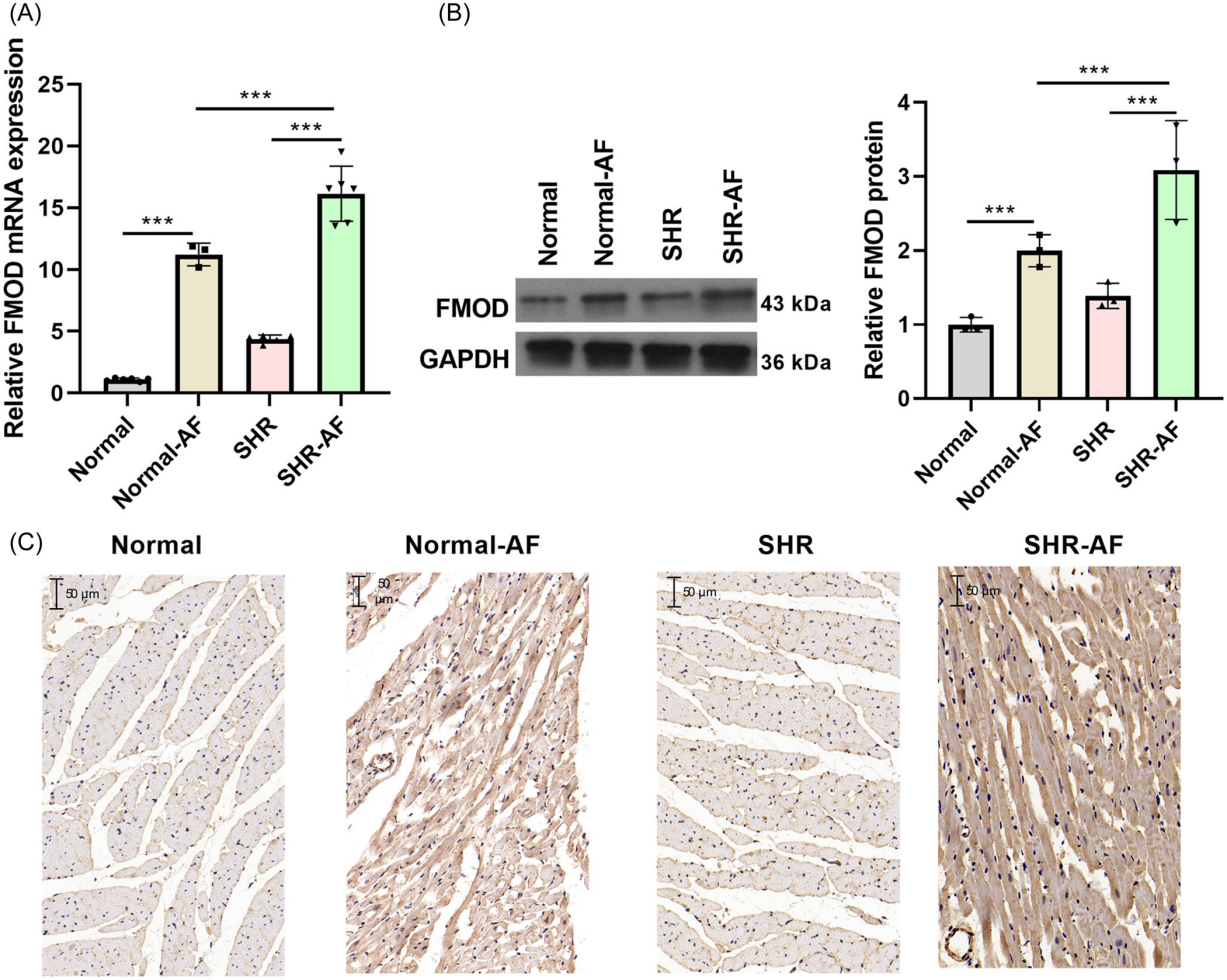
Expression levels of fibromodulin (FMOD) in various rat groups. (A) The relative mRNA expression levels of FMOD in atrial tissues of rats were measured by reverse transcription polymerase chain reaction (RT‐qPCR) 1 week after atrial fibrillation (AF) establishment. Normal group: *n* = 7; Normal‐AF group: *n* = 3; Spontaneously hypertensive rats (SHR) group: *n* = 5; SHR‐AF group: *n* = 6. (B, C) Protein expression levels of FMOD in atrial tissues of rats were measured using Western blot analysis and immunohistochemistry (IHC) 1 week after AF establishment. *n* = 3. ***p < .001.

The role of FMOD was investigated in the SHR‐AF animal model. AF was successfully induced in 26 of 36 SHR (72.2%), whereas only 3 out of 10 normal WKY rats (30%) developed AF, indicating SHRs' higher susceptibility to AF. One week after AF induction, FMOD relative mRNA and protein expression levels were notably upregulated in atrial tissues of both AF groups (normal‐AF and SHR‐AF groups) than that in atrial tissues of the no‐AF group (normal and SHR group). This effect was particularly pronounced in the SHR‐AF group (Figure 1). These findings suggest that FMOD may be involved in the development of AF in SHR.

### 3.2 FMOD expression was inhibited by sh‐FMOD infection in fibroblasts cells

**Figure 2:**
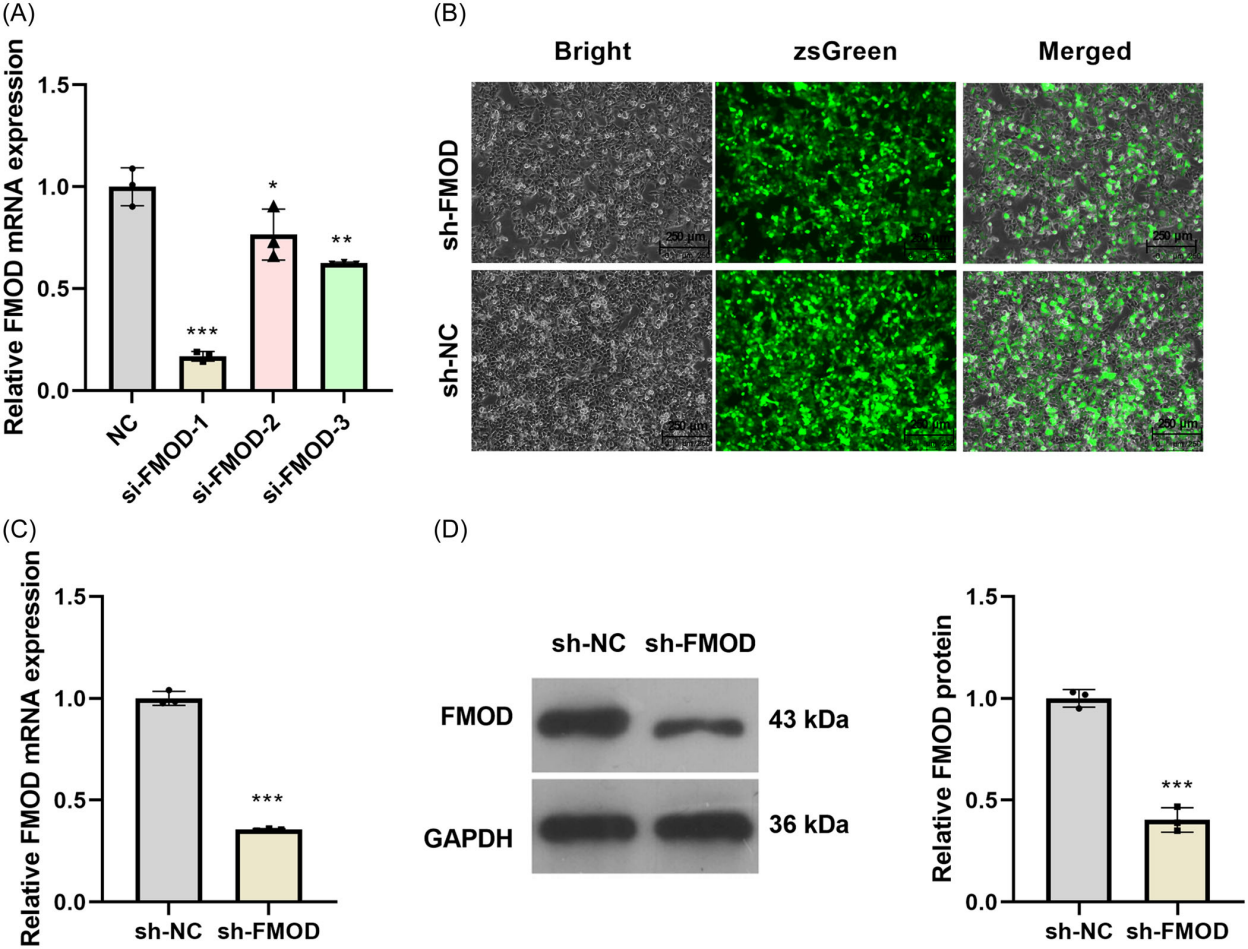
Fibromodulin (FMOD) expression was downexpressed in fibroblasts. (A) The relative mRNA expression levels of FMOD in fibroblasts after transfection with si‐FMOD were measured by RT‐qPCR. (B) GFP expression in fibroblasts visualized under a fluorescence microscope 24 h after infection with sh‐FMOD or sh‐NC. (C) FMOD mRNA expression in sh‐FMOD and sh‐NC groups was measured by RT‐qPCR. (D) FMOD protein level in sh‐FMOD and sh‐NC groups was measured by Western blot analysis. *p < .05, **p < .01, ***p < .001.

FMOD expression in fibroblasts was knocked down by transfection with FMOD siRNA (si‐FMOD). Three si‐FMOD sequences were selected. The mRNA expression of FMOD in fibroblasts was downregulated relative to that in the control after transfection with all three siRNA sequences, with si‐FMOD‐1 being significantly more efficient than si‐FMOD‐2 and si‐FMOD‐3 (Figure 2A). Thus, si‐FMOD‐1 was selected to construct an adenovirus‐mediated shRNA targeting FMOD (sh‐FMOD) to silence FMOD expression in fibroblasts. The results evidenced that GFP expression was enhanced 24 h after sh‐FMOD transduction, suggesting that adenoviruses successfully infected the fibroblasts (Figure 2B). Furthermore, FMOD mRNA expression and protein levels in the sh‐FMOD group were significantly higher than those in the sh‐NC group (Figures 2C,D).

### 3.3 Downregulation of FMOD expression in the SHR‐AF model

**Figure 3:**
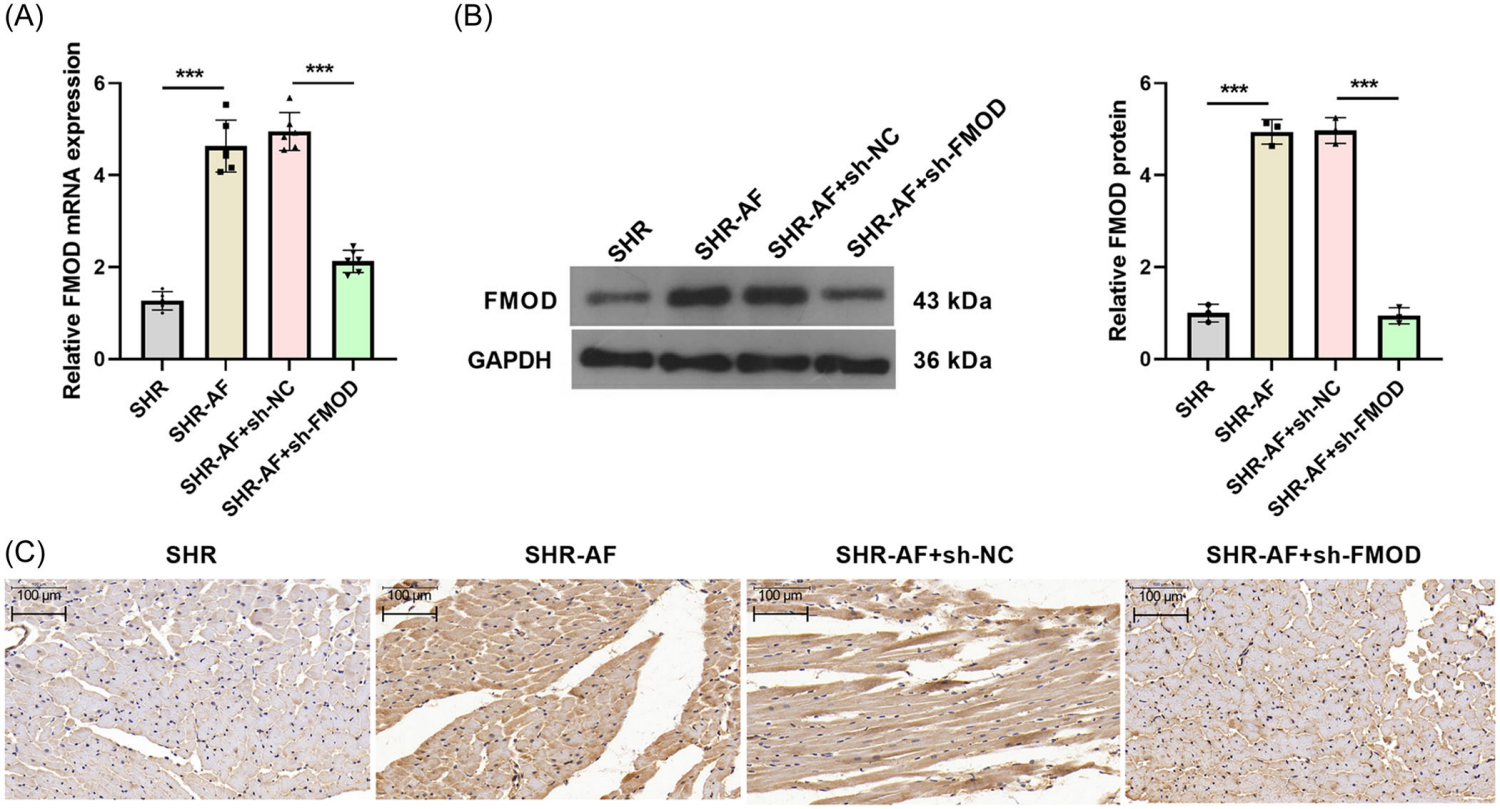
Fibromodulin (FMOD) expression was reduced in the SHR‐AF model 8 weeks after sh‐FOMD adenovirus injection. (A) The relative mRNA expression levels of FMOD in atrial tissues of rats was measured by reverse transcription polymerase chain reaction (RT‐qPCR) 8 weeks after adenovirus injection. Spontaneously hypertensive rats (SHR): *n* = 5; SHR‐AF, SHR‐AF + sh‐NC, and SHR‐AF+sh‐FMOD groups: *n* = 6. (B and C) The protein expression levels of FMOD in atrial tissues of rats were measured by Western blot analysis and immunohistochemistry (IHC) 8 weeks after adenovirus injection. *n* = 3. (***p < .001).

The sh‐FMOD adenovirus was used to silence FMOD expression in the SHR‐AF model. The mRNA and protein expression levels of FMOD in atrial tissues of the SHR‐AF group were higher than those in the SHR group (Figure 3). In addition, compared to the SHR‐AF + sh‐NC group, the mRNA and protein expression levels of FMOD were notably downregulated in the atrial tissues of the SHR‐AF + sh‐FMOD group 8 weeks after sh‐*FOMD* adenovirus injection (Figure 3).

### 3.4 Pathological atrium changes in the SHR‐AF model with downregulated FMOD expression

**Figure 4:**
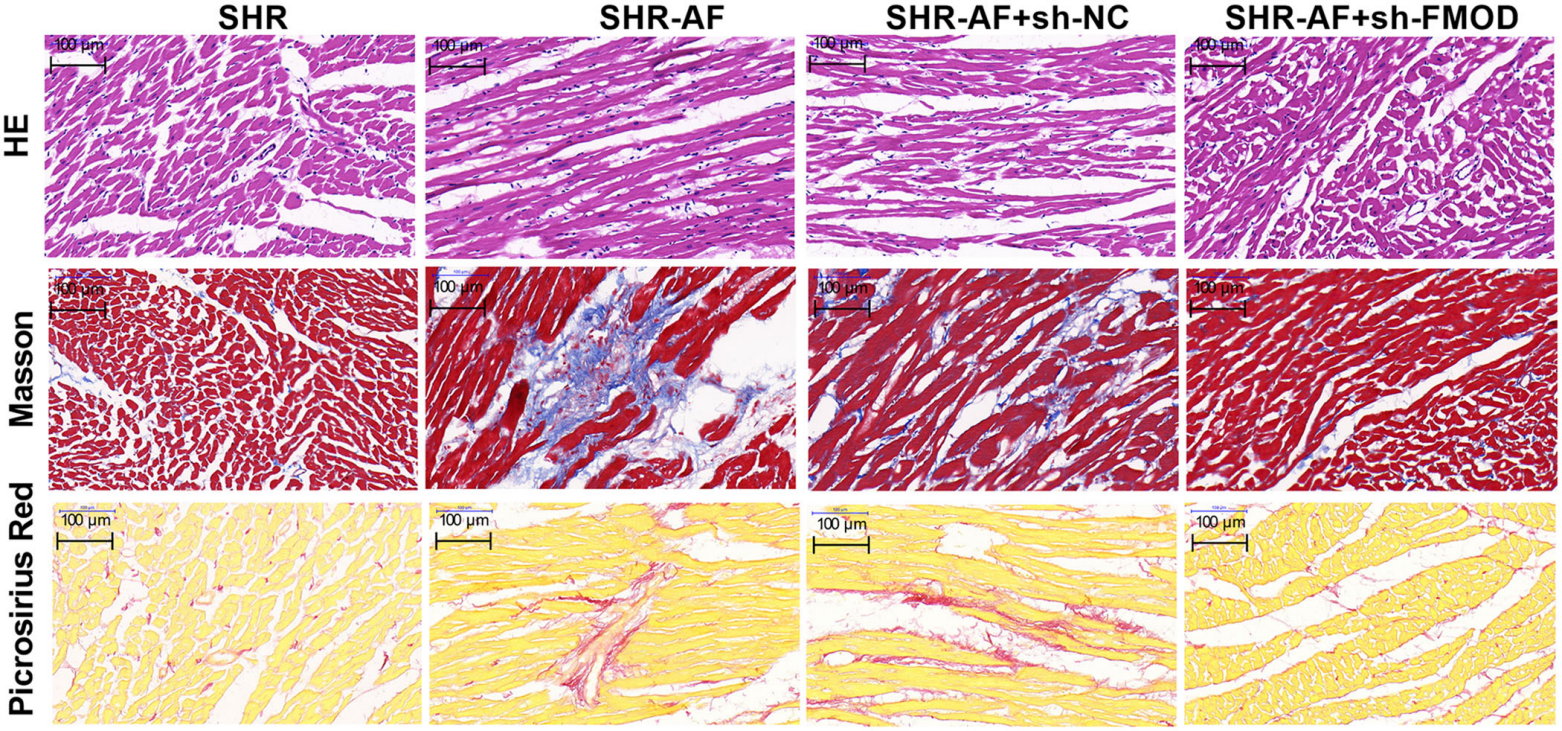
Fibromodulin (FMOD) knockdown can improve the pathology in SHR‐AF rats. Pathological changes in the atrium were assessed using hematoxylin and eosin (H&E), Masson's, and Picrosirius red staining 8 weeks after sh‐FOMD adenovirus injection.

**Figure 5:**
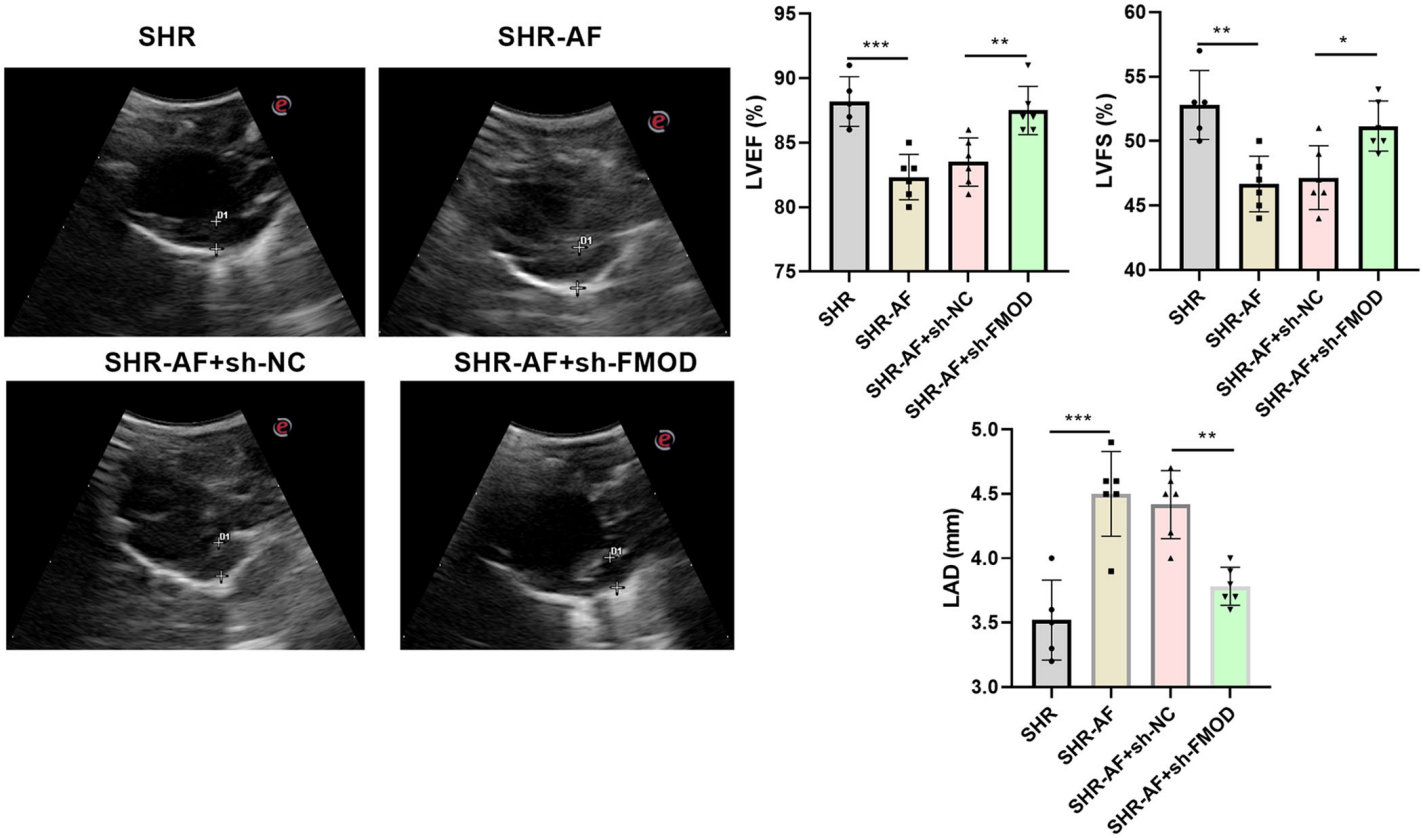
Fibromodulin (FMOD) knockdown can improve the cardiac function in SHR‐AF rats. Schematic diagram of echocardiography results 8 weeks after sh‐FOMD adenovirus injection. Representative images of the echocardiographic measurement are shown. Left atrial diameter is presented as the mean ± standard deviation. Spontaneously hypertensive rats (SHR): *n* = 5; SHR‐AF, SHR‐AF + sh‐NC, and SHR‐AF + sh‐FMOD groups: *n* = 6. *p < .05, **p < .01, ***p < .001.

Hypertension is an independent risk factor for atrial structural remodeling. H&E staining of atrial slices evidenced a notably increased extracellular matrix in atrial tissues from the SHR‐AF group compared to that in atrial tissues from the SHR group, whereas the extracellular matrix was notably decreased in atrial tissues from the SHR‐AF(+sh‐FMOD) group compared to that in atrial tissues from the SHR‐AF(+sh‐NC) group (Figure 4). Masson's staining of atrial slices showed that extracellular matrix collagen was notably increased in atrial tissues from the SHR‐AF group compared to that in those from the SHR cohort, whereas the extracellular matrix was notably decreased in atrial tissues from the SHR‐AF(+sh‐FMOD) group compared to that in those from the SHR‐AF(+sh‐NC) group. Picrosirius red staining showed that collagen fibers were notably increased in atrial tissues from the SHR‐AF group compared to that in those from the SHR group, whereas the extracellular matrix was notably decreased in atrial tissues from the SHR‐AF(+sh‐FMOD) group compared to that in those from the SHR‐AF(+sh‐NC) group. Additionally, echocardiography results showed that the LAD in the SHR‐AF group was notably higher than that in the SHR group, whereas the LVFS and LVEF were notably lower (Figure 5). FOMD knockdown can improve the LAD, LVFS, and LVEF in SHR‐AF rats (Figure 5).

### 3.5 Downregulation of FMOD suppressed the inflammasome expression and myocardial fibrosis in the SHR‐AF model

**Figure 6:**
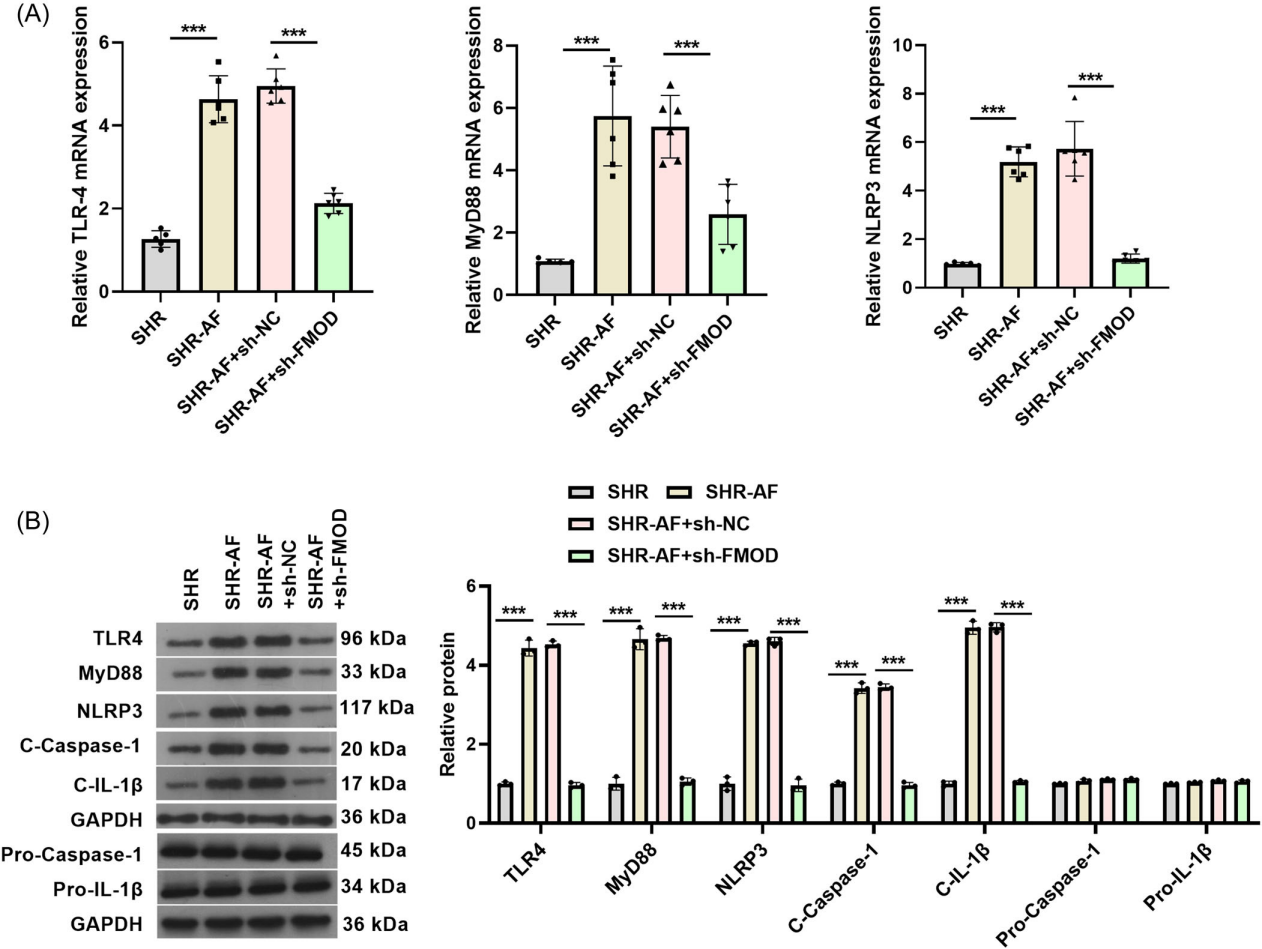
Fibromodulin (FMOD) knockdown can silence the TLR4/MyD88/NLRP3 in SHR‐AF rats. (A) Expression of TLR4, MyD88, and NLRP3 mRNAs in atrial tissues were measured via RT‐qPCR 8 weeks after sh‐FOMD adenovirus injection. Spontaneously hypertensive rats (SHR): *n* = 5; SHR ‐AF, SHR‐AF + sh‐NC, and SHR‐AF + sh‐FMOD groups: *n* = 6. (B) Protein expression levels of TLR4, MyD88, NLRP3, cleavage‐caspase‐1, and cleavage‐IL‐1β in atrial tissues were measured via western blot analysis 8 weeks after sh‐FOMD adenovirus injection. *n* = 3. ***p < .001. C‐Caspase‐1, Cleavage‐Caspase‐1; C‐IL‐1β, Cleavage‐IL‐1β.

**Figure 7:**
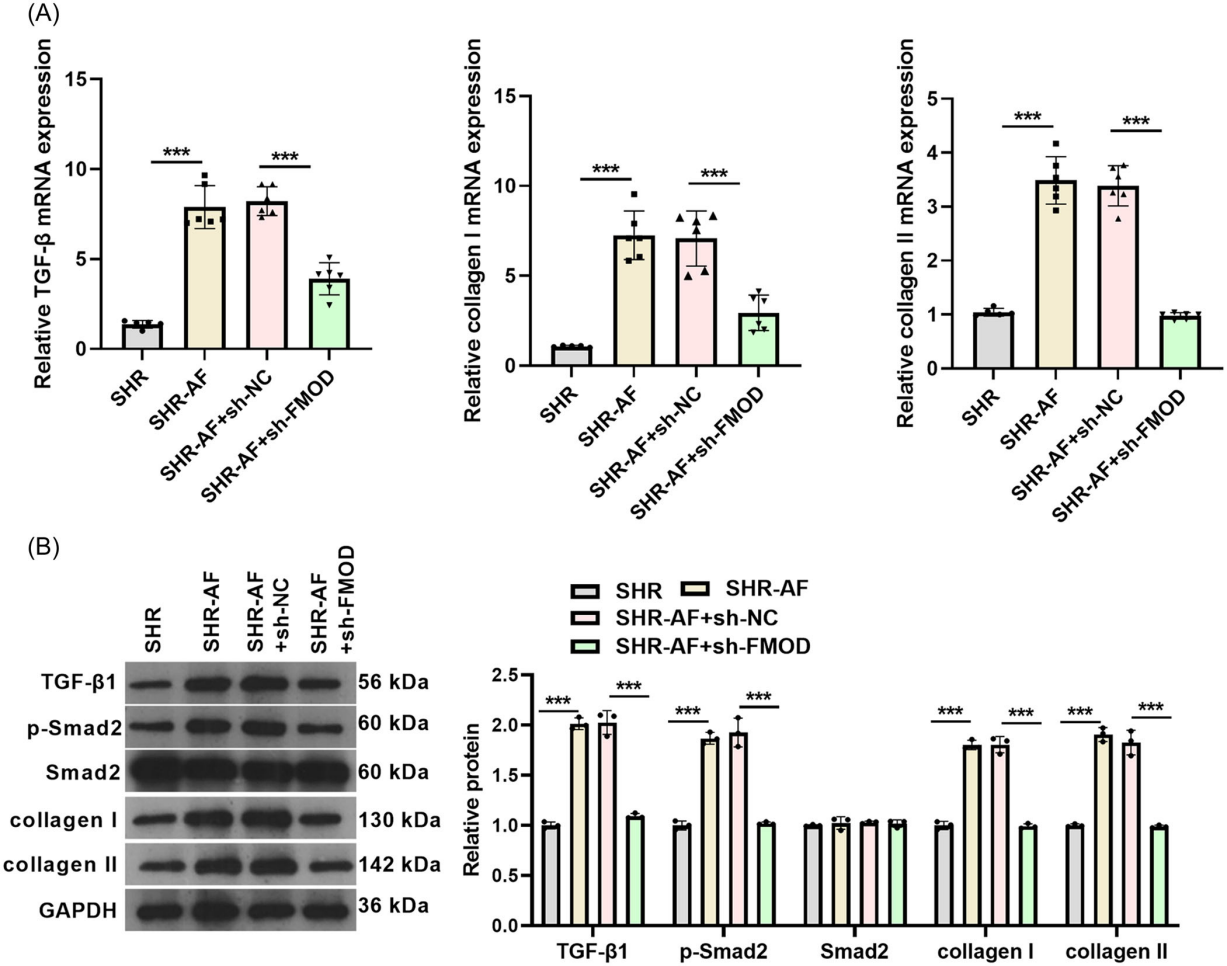
FOMD knockdown can silence the TGF‐β1/Smad and inhibit fibrosis in SHR‐AF rats. (A) Expression of TGF‐β1, collagen I, and collagen II mRNAs in atrial tissues were measured via RT‐qPCR 8 weeks after sh‐FOMD adenovirus injection. SHR: *n* = 5; SHR‐AF, SHR‐AF + sh‐NC, and SHR‐AF + sh‐FMOD groups: *n* = 6. (B) Protein expression levels of TGF‐β1, p‐Smad2, Smad2, collagen I, and collagen II in atrial tissues were measured via Western blot analysis 8 weeks after sh‐FOMD adenovirus injection. *n* = 3. ***p < .001.

RT‐qPCR evidenced that mRNA of TLR4, MyD88, and NLRP3 were markedly increased in atrial tissues from the SHR‐AF group rats compared to that in those from the SHR group, whereas these mRNA were notably decreased in atrial tissues from the SHR‐AF(+sh‐FMO*D*) group compared to that in those from the SHR‐AF(+sh‐*NC*) group (Figure 6A). Western blot analysis evidenced that protein levels of TLR4, MyD88, NLRP3, Cleavage‐caspase‐1, and Cleavage‐IL‐1β were markedly increased in atrial tissues from the SHR‐AF group rats compared to that in those from the SHR group, whereas these protein levels were notably decreased in atrial tissues from the SHR‐AF(+sh‐FMO*D*) group compared to that in those from the SHR‐AF(+sh‐*NC*) group (Figure 6B). RT‐qPCR evidenced that mRNA of TGF‐β1, collagen I, and collagen II were markedly increased in atrial tissues from the SHR‐AF group rats compared to that in those from the SHR group, whereas these mRNA were notably decreased in atrial tissues from the SHR‐AF(+sh‐FMO*D*) group compared to that in those from the SHR‐AF(+sh‐*NC*) group (Figure 7A). In addition, the protein expression of fibrotic biomarkers including TGF‐β1, p‐Smad2, collagen I, and collagen II were markedly increased in atrial tissues from the SHR‐AF group compared to that in those from the SHR group, whereas these protein levels were notably decreased in atrial tissues from the SHR‐AF(+sh‐FMOD) group compared to that in those from the SHR‐AF(+sh‐*NC*) group (Figure 7B).

## 4 DISCUSSION

This study indicated that FMOD expression was upregulated in the atrial tissues of both the SHR and SHR‐AF groups, with a particularly significant increase in the SHR‐AF group. This suggests that FMOD may be involved in SHR‐AF development. The extracellular matrix and collagen fibers were notably enriched in atrial tissues from the SHR‐AF group compared to that in atrial tissues from the SHR group, whereas knockdown of FMOD in SHR‐AF atrial tissues decreased the extracellular matrix, extracellular matrix collagen, and collagen fibers. Moreover, we found that the downregulation of FMOD suppressed the TLR4/MyD88 signaling pathway, inflammasome, and fibrotic biomarkers in the SHR‐AF atrial tissues. These findings indicate that the downregulation of FMOD could attenuate inflammatory signaling and atrial fibrosis in SHR‐AF.

Hypertension is an important factor affecting cardiovascular diseases, especially the development of AF.^26^ FMOD contributes to the formation of collagen fibrils and the extracellular matrix.^13^ Previous studies have evidenced that FMOD expression was upregulated during lung fibrosis and wound healing processes in the skin.^27^, ^28^ Moreover, Andenæs et al.^23^ reported that FMOD expression was enhanced in patients with heart failure. However, its effect in atrial tissues of SHR and SHR‐AF remains unclear. Our results evidenced that FMOD was upregulated in the atrial tissues of SHR and SHR‐AF, especially in SHR‐AF, suggesting that FMOD plays a role in AF.

FMOD modulates extracellular matrix organization by interacting with collagen I and collagen II,^29^ reprogramming human fibroblasts^30^ and collagen fibrillogenesis.^31^ Fibrillar collagen accumulated in the extracellular matrix can promote cardiac fibrosis.^32^ In this study, FMOD was silenced by RNA interference to investigate the role of FMOD in SHR‐AF. We found that fibrotic features, including the extracellular matrix, collagen, and collagen fibers, were notably enriched in atrial tissues from the SHR‐AF group than in those from the SHR group, whereas downregulation of FMOD expression decreased the extracellular matrix, collagen, and collagen fibers in atrial tissues from SHR‐AF. This indicates that hypertension was accompanied by AF‐induced cardiac fibrosis, whereas the downregulation of FMOD expression attenuated atrial fibrosis features in SHR‐AF. A larger LAD is related to AF occurrence and recurrence and is an independent predictor of cardiovascular disease in patients with AF.^33^, ^34^, ^35^ LAD was clearly enhanced in patients with hypertension.^36^ In this study, we found that the LAD was significantly larger in the SHR‐AF group than in the SHR group. In addition, Both of LVFS and LVEF represent the systolic function of the heart, and the higher these two indicators, the better the left ventricular systolic function of the heart. Silencing FMOD expression decreased the LAD and increased the LVFS and LVEF, suggesting that reduced FMOD expression can ameliorate AF occurrence and recurrence and improve the cardiac function.

TLR4 mediates inflammatory signaling pathways in myocardiac diseases.^37^, ^38^, ^39^ TLR4/myeloid differentiation factor 88 (MyD88) signaling can activate the NLRP3 inflammasome and proinflammatory factors, leading to myocardial injury.^40^, ^41^, ^42^ The NLRP3‐inflammasome is increased in atrial cardiomyocytes and drives caspase‐1 activation and interleukin‐1β release to promote cardiomyocyte remodeling and induces AF pathogenesis.^43^, ^44^, ^45^ A previous study reported that IL‐1β promotes atrial hypertrophy and AF development.^46^ In this study, we found that the protein levels of TLR4, MyD88, NLRP3, caspase‐1, and IL‐1β in atrial tissues were markedly increased in the SHR‐AF group compared to those in the SHR group, whereas downregulation of FMOD decreased the protein levels in atrial tissues of the SHR‐AF group. These findings indicate that the downregulation of FMOD could attenuate inflammatory responses in the atrial tissues in SHR‐AF. TGF‐β1 is a key mediator of fibrosis, and pro‐fibrotic collagen I and collagen II are fibrotic biomarkers. He et al.^47^ reported that the upregulation of TRPC3 via TGF‐β1/Smad2/3 regulates atrial fibrosis in SHR and during aging. Similar to these results, we found that the protein levels of TGF‐β1, p‐Smad2, collagen I, and collagen II were markedly increased in SHR‐AF compared with those in SHR, whereas downregulation of FMOD decreased them. These findings indicate that the downregulation of FMOD attenuated fibrosis in the atrial tissues of SHR‐AF.

This study had some limitations. First, although we characterized the effects of FMOD in an animal model of SHR‐AF for the first time, we did not verify FMOD expression in patients with SH‐AF. Second, the downregulation of FMOD attenuated inflammatory signaling and fibrosis in the atrial tissues of SHR‐AF, but the exact mechanisms involved were not elucidated. Further studies are required to elucidate the association between FMOD and SHR‐AF. FMOD is expressed in various organs and tissues, including the arteries, pancreas, and bones. When adenovirus‐mediated shRNA targeting FMOD was injected into the tail vein, we could not guarantee that it was specifically targeting the heart tissue; therefore, we cannot exclude its side effects or other influencing factors on other organs. Finally, the AF model was induced by rapid transesophageal AF, and the success rate was 72.2% in SHR and only 30% in normal WKY rats. However, a previous study found that the success rate of an injection‐induced AF model reached 90% in male Wistar rats.^48^ Hence, in future studies, the SHR‐AF model should be induced by injection.

## 5 CONCLUSION

This study demonstrated that FMOD was upregulated in the atrial tissues of the SHR and SHR‐AF groups, particularly in the SHR‐AF group. Downregulation of FMOD attenuated inflammatory signaling and fibrosis in atrial tissues of SHR‐AF. Thus, FMOD may be a promising therapeutic target for the treatment of spontaneous hypertension in patients with AF.

## AUTHOR CONTRIBUTIONS

Yuming Liang and Yan He conceived and designed the study and developed the methodology. Yuming Liang, Yun Zhou, and Jialin Wang performed the experiments and collected the data. Yuming Liang and Yun Zhou analyzed and interpreted the data. Yuming Liang drafted the manuscript. Yun Zhou and Yan He revised the manuscript. All authors have read and approved the final version of the manuscript.

## CONFLICT OF INTEREST STATEMENT

The authors declare no conflict of interest.

## ETHICS STATEMENT

All animal experiments were performed in accordance with the National Institutes of Health Guidelines on the Use of Laboratory Animals and approved by the Animal Care and Welfare Committee of Guangxi Medical University (Approval No. 201807072).

## Data Availability

The datasets analyzed in the current study are available from the corresponding author upon request.
